# Characterizing Key Volatile Pollutants Emitted from Adhesives by Chemical Compositions, Odor Contributions and Health Risks

**DOI:** 10.3390/molecules27031125

**Published:** 2022-02-08

**Authors:** Zixuan Zhao, Yipu Pei, Peng Zhao, Chuandong Wu, Chen Qu, Weifang Li, Yanjun Zhao, Jiemin Liu

**Affiliations:** 1 School of Chemistry and Biological Engineering, University of Science and Technology Beijing, Beijing 100083, China; zzxmht@163.com (Z.Z.); wuchuandong@ustb.edu.cn (C.W.); quchen5626@163.com (C.Q.); zhaoyj@ustb.edu.cn (Y.Z.); 2 State Environmental Protection Key Laboratory of Odor Pollution Control, Tianjin Academy of Ecoenvironmental Sciences, Tianjin 300191, China; lwf1919@163.com; 3 China Building Material Test & Certification Group Co., Ltd., Beijing 100024, China; peiyipu@ctc.ac.cn; 4 Beijing Municipal Institute of Labor Protection, Beijing 100054, China; zhaopeng@bmilp.com

**Keywords:** indoor air, building materials, adhesive, odor, volatile organic compounds, health risk

## Abstract

As one of the major sources of volatile pollutants in indoor air, gaseous emissions from adhesives during interior decoration have attracted increasing concern. Identifying major volatile pollutants and the risk in adhesive gaseous emissions is of great significance, but remains rarely reported. In the present research, we assessed the major volatile pollutants emitted from white emulsion adhesive and silicone adhesive samples (n = 30) from three aspects: chemical composition, odor and health risk contributions. The results showed that a total of 21 volatile pollutants were detected. Significantly, xylene was the most concentrated compound from white emulsion adhesives, accounting for 45.51% of the total concentrations. Butanone oxime was the most concentrated compound in silicone adhesives, accounting for 69.86% of the total concentrations. The trends in odor concentration (evaluated by the odor activity value method) over time were well correlated with the total chemical concentrations. Xylene (58.00%) and butanone oxime (76.75%) showed the highest odor contribution, respectively. Moreover, from an integrated perspective of chemical emissions, odor and health risk contributions, xylene, ethylbenzene, ethyl acetate and benzene were identified as the key volatile pollutants emitted from the white emulsion adhesives, while butanone oxime, butanone, and ethanol were the key volatile pollutants emitted from the silicone adhesives. This study not only identified the key volatile pollutants but also provided characteristics of odor and health risks of gas emitted from adhesives.

## 1. Introduction

In the area of indoor air quality, studies on gaseous emissions from building materials are drawing increasing attention. During the decoration process, building materials such as adhesives emit large amounts of volatile organic compounds (VOCs) [1,2,3,4,5], which are a major source of indoor air pollution. Since people spend approximately 80% of their lifetime in indoor buildings [6,7], long-term exposure to these VOCs can cause serious odor nuisance effects and negative health effects, leading to sick building syndrome (SBS) [8,9,10,11].

The most studied VOCs emitted from building materials include aromatic compounds, aldehydes, and *n*-hexane, which are major indoor air pollutants due to their malodorous and hazardous properties [12,13,14,15,16]. Long-term exposure to these VOCs can cause odor nuisances such as anxiety, headache, nausea, and severe damage to respiratory and nervous systems [17]. Building materials, wooden flooring and furniture were identified as the main sources of residential indoor VOCs [18,19,20]. Jiang et al. [21] revealed that aldehydes, especially hexanal and pentanal, were dominant odorous pollutants emitted from particleboard due to their low odor thresholds. Liang [22] pointed out that octanal was the main odor contributor during house renovation. Moreover, the health risks were used to assess the impact of hazardous gas on people [23]. Fang et al. [24], Zhang et al. [25], Masih et al. [26] and Hadei et al. [27] revealed that the health risks of formaldehyde, BTEX, etc., exceed acceptable levels in indoor air. The odor nuisances and hazards of VOCs in indoor environments are not only due to their volatility but also to their wide use in household products [28,29,30,31]. For example, Marchand et al. [32] found that the main sources of indoor aldehydes included hardwoods, plywood, laminate flooring, etc. Jiang, Li, Zhang, Li, Wang and Yu [21] found that the emissions of HCHO and 44 VOCs were identified with their dependence on temperature, while *n*-hexane and formaldehyde were most concentrated among the 45 compounds detected from particleboard.

In modern architecture, various adhesives are widely used to paste decorative materials and furniture during indoor decoration. Adhesives are applied underneath decorative materials and are usually out of sight, so their impact on indoor air quality is easily ignored. It is worth noting that adhesives contain abundant organic solvents [33,34,35], which could be the most dominant contributor to indoor VOCs in some cases [36,37,38]. Moreover, previous studies have mainly focused on the chemical compositions, emission factors and rates of VOC emissions [34,39,40,41], integrated investigations on the odor and health risks of VOC emissions from adhesives and other building materials have rarely been addressed. Therefore, it is important to analyze the odor pollution and health risk contributions from adhesives to identify the key volatile pollutants and provide informative strategies to precisely reduce the related odor nuisances and health risks in indoor air.

In the current study, we present a comprehensive evaluation of the chemical compositions, odor and health risk contributions of volatile compounds emitted from 30 adhesives that are commonly available on the market. Both instrumental and sensory methods were applied for odor evaluation. Trends in concentrations and odor potentials along with the emission time were evaluated, and the correlation between the concentrations and odor concentration was calculated. The key volatile pollutants emitted from adhesives were identified based on their contributions to the overall odor and health risk contributions.

## 2. Results and Discussion

### 2.1. Identification of VOCs Emitted from the Adhesives

**Figure 1:**
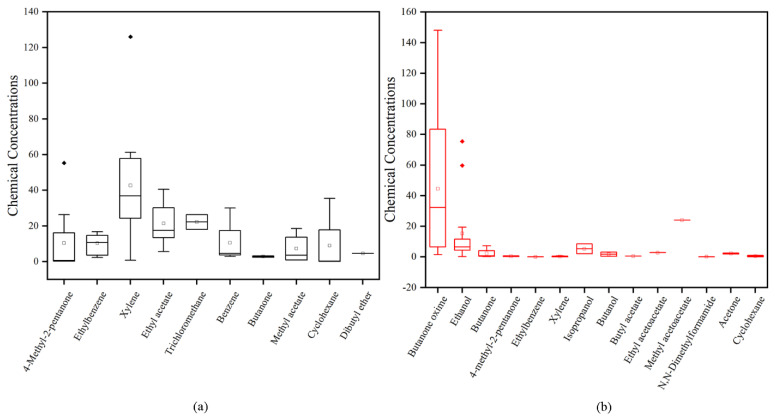
Box diagram of concentration distributions of the compounds emitted from white emulsion adhesives (**a**) and silicone adhesives (**b**).

Table 1 summarizes the detection compounds. In this study, a total of 21 volatile compounds were detected in the gas emitted from the adhesive samples, and their chemical concentrations were quantified. As shown in Table 1, the most common substances were 4-methyl-2-pentanone, ethylbenzene, xylene, butanone oxime, butanone, ethanol, cyclohexane, etc. The detection frequencies of 4-methyl-2-pentanone, xylene and butanone oxime were 100% in white emulsion adhesives and silicone adhesives, respectively. Among these VOCs, xylene (max: 125.99 mg/m^3^) and butanone oxime (max: 1036.71 mg/m^3^) had the highest concentrations in the white emulsion adhesives and silicone adhesives, respectively. Figure 1 shows the concentration distributions frequencies, chemical concentrations and odor threshold values of these detected of each compound detected in a visual way.

#### 2.1.1. White Emulsion Adhesives

In decoration, white emulsion adhesive is one of the most versatile, most used and oldest water-soluble adhesives. White emulsion adhesive is a thermoplastic adhesive prepared by the polymerization of vinyl acetate monomer under the action of an initiator, with the advantages of room temperature curing, relatively fast curing and high adhesive strength. In this study, 11 volatile organic compounds, including ketones, esters, benzene, alcohols (ethers) and alkanes, were quantified in the gas emitted from the 10 white emulsion adhesive samples, and the results are shown in Table 1. Xylene was dominant in these samples, with an average concentration of 42.70 mg/m^3^, and the maximum concentration was 125.99 mg/m^3^. The average concentrations of trichloromethane, ethyl acetate, benzene, 4-methyl-2-pentanone and ethylbenzene decreased in order, with 22.23 mg/m^3^, 21.47 mg/m^3^, 10.59 mg/m^3^, 10.36 mg/m^3^ and 10.27 mg/m^3^, respectively. Xylene and 4-methyl-2-pentanone were detected in every sample. The detection frequencies of ethylbenzene and methyl acetate were 70%, but their average concentrations were only 0.17–0.24 times that of xylene. The overall concentration contribution of these VOCs is shown in Figure 1a. Due to its large concentration advantage, xylene had an average concentration contribution of 45.51%.

#### 2.1.2. Silicone Adhesives

**Figure 2:**
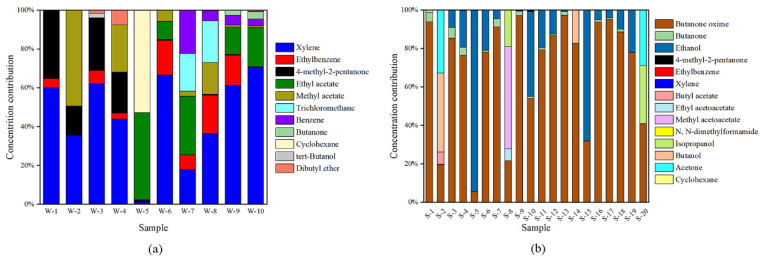
The concentration contribution of volatile compounds emitted from white emulsion adhesives (**a**) and silicone adhesives (**b**).

Silicone adhesive is a material that resembles ointment, and it cures into a tough rubber-like solid once it contacts moisture in the air. Silicone adhesive is often used for bonding and sealing in glass and has excellent properties of aging resistance, good elasticity, outstanding weather resistance and UV resistance. As the main source of indoor VOCs, silicone adhesives cannot be ignored, especially in newly built buildings. In this study, a total of 14 compounds were quantified in the emitted gases of 20 silicone adhesives, and the concentrations are shown in Table 1. The detected compounds included ketones, alcohols, benzenes, esters and nitrogenous organic compounds. The representative compounds that we identified were butanone oxime, butanone and ethanol, all of which had high chemical concentrations and detection frequencies. The average concentrations in descending order were butanone oxime (668.34 mg/m^3^) > ethanol (231.02 mg/m^3^) > butanone (33.17 mg/m^3^), with corresponding detection frequencies of 100%, 70% and 65%, respectively. The concentration contributions of butanone oxime, butanone and ethanol in gaseous emissions from each silicone adhesive sample were also studied (Figure 2b). Butanone oxime was found in every emitted gas and dominated the concentrations since it was added to the silicone adhesive as a curing agent for silicon. The average concentration contribution of butanone oxime was 69.86% and was greater than 50% in 15 silicone adhesive samples. This result might be due to most of the studied adhesives being of the deketoxime neutral variety, which could release butanone oxime during the curing process. This kind of adhesive is noncorrosive to aluminum, coated glass, and inorganic materials but corrosive to copper, lead, zinc, and polycarbonate. Jarnstrom et al. [42] also revealed that the main VOCs emitted from neutral silicone adhesives in new residential construction in Finland was butanone oxime. Ethanol was determined in 14 silicone adhesive samples, and its concentration accounted for 23.85% of the total emitted gas concentration on average (maximum of 94.31%). Ethanol might be emitted from dehydrated neutral silicone adhesives, which are more widely used. Dehydrated neutral silicone adhesives release small molecular weight alcohols during curing and are harmless to any coating material. It is worth noting that butanone had a higher detection frequency than ethanol, while its average concentration was much lower than that of butanone and butanone oxime. The reason might be that butanone is a common solvent in silicone adhesives [43].

**Figure 3:**
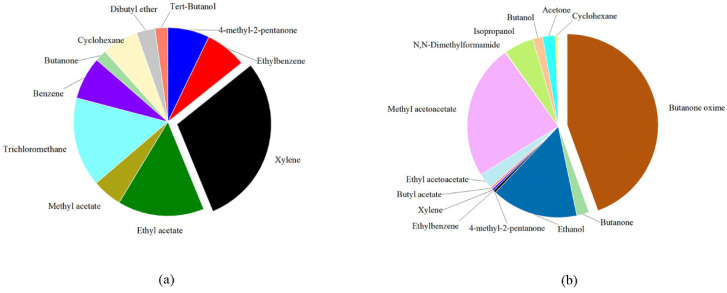
Contributions to cumulative average concentrations of the compounds emitted from white emulsion adhesives (**a**) and silicone adhesives (**b**).

The types and properties of adhesives vary depending on the substances they contain. As two commonly used adhesives, white emulsion and silicone adhesives release different compounds. Figure 3 shows contributions to cumulative average concentrations of the compounds emitted from adhesive samples. The relative proportion of each compound is clearly visible in this figure. It is obvious that xylene and butanone oxime dominate the concentrations of white emulsion and silicone adhesives, respectively. The detection frequencies of trichloromethane and benzene are not greater than 40%, but their cconcentrations are considerable. In association with the health risk potentials in Section 2.3.2, trichloromethane and benzene should be of sufficient concern. Compared with white emulsion adhesives, the compositions and amounts of silicone adhesives are completely different. In addition to butanone oxime, ethanol also has a significant share, which may prove that deketoxime and dehydration are the two main forms present in silicone adhesives. 

**Figure 4:**
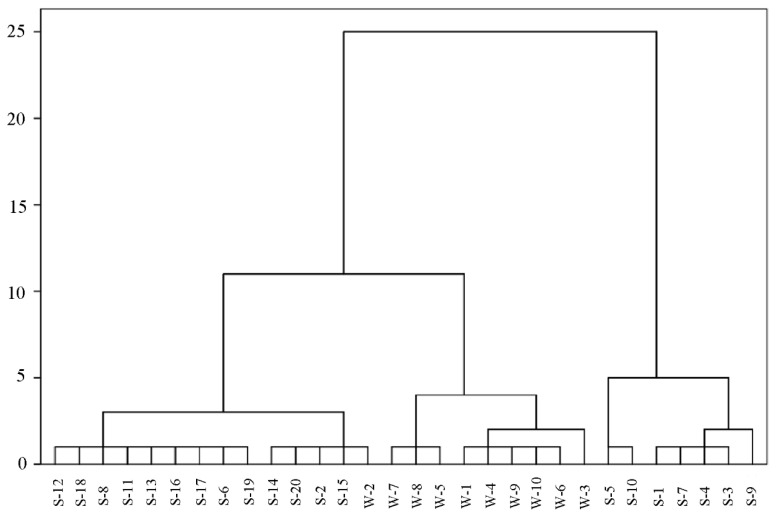
Dendrogram of adhesive samples.

Furthermore, cluster Analysis was performed to distinguish adhesive samples according to the compounds and their related concentrations, the results of which are presented in Figure 4. White emulsion adhesives and silicone adhesives could be roughly clustered into three groups, two of which are silicone adhesives (S-12–S-15, S-5–S-9) and the other being white emulsion adhesives (W-7–W-3). Adhesives with the same characteristics are clustered into one group. For example, W-7 and W-8 were grouped together due to the existence of trichloromethane. S-5–S-9 were put together as a result of the concentrations of butanone oxime being higher than other silicone adhesives.

### 2.2. Trends in Concentrations and Odor Potentials with the Pre-Emission Time

VOCs produced by adhesives are one of the main sources of indoor air pollutants and can also easily cause odor pollution. To simulate the variation in VOC emissions from adhesives with time after being coated, the effect of pre-emission time on the concentrations and odor potentials of the emitted volatile compounds were further investigated. Two samples (W-7, S-18) were selected from the investigated white emulsion and silicone adhesives to explore the trends in total chemical concentrations and odor concentration (evaluated by the *SOAV*) with the pre-emission time. We extended the pre-emission time (by an additional 15, 30, 45, 60 and 90 min) before placing the samples in the emission chamber to simulate different emission times of adhesives In indoor air. The total chemical concentrations of volatile compounds emitted from these two samples were determined by the same methods as those for the previous 30 samples. Odor threshold (*C_OT_*) values were used to convert the chemical concentrations into *OAVs* and *SOAVs* according to Equation (2) (Section 3.4).

**Figure 5:**
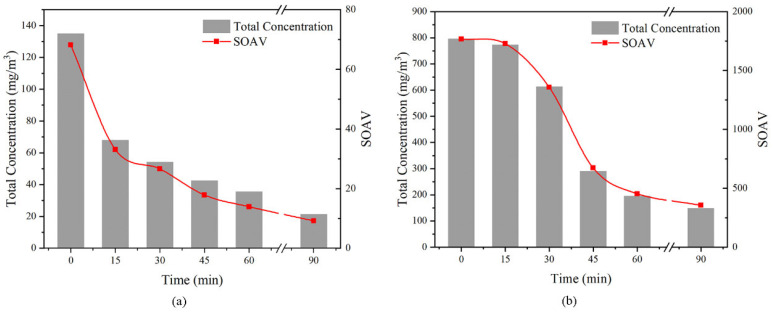
Trends in concentrations and odor potentials with the pre-emission time of white emulsion adhesive (**a**) and silicone adhesive (**b**).

Figure 5 shows the changes in total concentrations and *SOAV* with time of white emulsion adhesive (a) and silicone adhesive (b). The chemical concentrations of the emitted volatile compounds showed clear decreasing trends with extension of the pre-emission time. In white emulsion adhesives, the total concentration decreased fastest within 15 min. The total concentrations decreased to half in 15 min and were approximately 15% after being emitted for 90 min. For the silicone adhesives, there were few changes in total concentrations in the first 15 min. The total concentrations decreased to less than 40% after being emitted for 45 min.

The trend in *SOAV* with pre-emission time was consistent with the total chemical concentrations. Statistical analysis showed that the *SOAV* was well correlated with the total chemical concentrations (r = 0.998, *p* < 0.01). The *SOAV* is frequently used as a surrogate of the odor concentration of gaseous mixtures to assess the strength of an odor [44,45]. The trends in *SOAV* with the emission time provided knowledge on the variation in odor potentials in gaseous emissions from these adhesives.

### 2.3. Identification of the Key Volatile Pollutants from the Perspectives of Odor Nuisances and Health Risks

#### 2.3.1. Odor Contribution of VOCs Emitted from the Adhesives

VOCs emitted from building materials are the main source of indoor odor pollution, while the odor contribution of these compounds may be disproportionate relative to their chemical concentrations since each compound has different odor properties and odor thresholds. The *OAV* and odor contribution (*P_i_*) are useful metrics to assess individual odor contributions. As a chemical concentration weighted by an individual odor threshold value, *OAV* better characterizes the odor properties than the chemical concentration and is used to estimate odor intensity, as well as to assess the odor contribution of individual compounds [44,46,47]. Thus, *OAV* and *P_i_* can be effectively used to identify key odorant substances from the perspective of contributions to the overall odor of air samples. Theoretically, compounds with *OAVs* less than 1 cannot be perceived by the general population, while compounds with *OAVs* over 1 are expected to contribute significantly to the perceived odor. The higher the *OAV* is, the stronger the odor produced by the compound [21].

**Figure 6:**
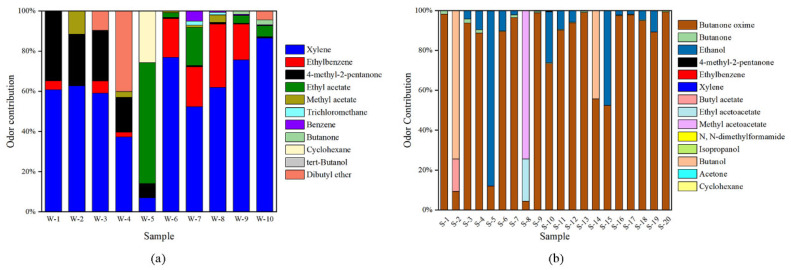
Odor contribution of volatile compounds emitted from white emulsion adhesives (**a**) and silicone adhesives (**b**).

Figure 6a shows the comparison of the odor contribution of each compound (detailed *OAVs* of each compound are listed in Table S1. The discrepancy in odor contributions is caused by both chemical concentration and odor threshold values. More specifically, xylene contributed most to the overall odor of gas emitted from white emulsion adhesives (except from sample S-5), and the odor contribution was greater than 60% in most of the samples due to its low odor threshold value and the dominating concentration proportion. Furthermore, although the concentrations of trichloromethane and benzene were not low, their odor contributions were insignificant owing to the large odor threshold values. The odor contribution of ethyl acetate in sample W-5 was obviously different from the other samples since the concentration of ethyl acetate was nearly 40 times higher than that of xylene and 4-methyl-2-pentanone. The average odor contributions of xylenes, ethyl acetate, ethylbenzene and 4-methyl-2-pentanone in the 10 white emulsion adhesive samples decreased by 58.00%, 18.17%, 14.50% and 11.30%, respectively.

The detailed *OAVs* of each compound in gaseous emissions from the 20 silicone adhesive samples were calculated, as shown in Table S2. Nearly all the detected compounds had *OAVs* greater than 1, except for ethylbenzene, xylene, isopropanol, acetone and cyclohexane, with *OAVs* less than 1 in four of the 20 samples. This result is mainly because the odor threshold values of these five compounds are quite large, and the chemical concentrations of ethylbenzene, cyclohexane and xylene in these samples were too low.

Figure 6b illustrates the odor contribution of volatile compounds in the emitted gas of each of the silicone adhesive samples. It is obvious that the odor contribution of butanone oxime dominates in most silicone adhesives (average of 76.75%), which makes this compound the main odor source of these adhesives. The odor contribution of butanone oxime in gaseous emissions from 11 silicone adhesive samples was greater than 90%, while the odor contributions of the remaining substances in these samples were mostly less than 5%. These results indicated that from the point of view of odor nuisance, butanone oxime is the main volatile pollutant in the gas emitted from silicone adhesives. On the other hand, the average odor contributions of butanone and ethanol, which also had high detection frequencies, were only 0.83% and 15.81%, respectively. Although butyl acetate, ethyl acetoacetate, methyl acetoacetate and butanol had significant odor contributions in isolated samples, their detection frequencies were only 5%, which was not representative.

#### 2.3.2. Health Risk Potentials

The above 30 adhesive samples showed wide difference in chemical compositions and concentrations. To further compare the health risk potentials caused by the compounds released from these adhesives, inhaled cancer and noncancer risks were calculated according to the methodology recommended by the United States Environmental Protection Agency (USEPA) [48,49]. The exposure concentration (EC) was estimated to calculate the inhalation exposure to volatile compounds emitted from adhesives. In the present study, the ECs were assessed based on the concentration of volatile compounds emitted from the testing chamber, and since the volatile compounds shared the same dispersion pathway and exposure patterns, these concentrations were proportional to the exposure concentrations in real indoor air when adhesives were applied. Hence, these values could provide knowledge of the comparison of the health risk potentials of gases emitted from the 30 adhesives. In addition, the proportions of HI (expressing the noncancer risk) and R (expressing the cancer risk) could be used to identify the key volatile pollutants from the perspective of health risks.

**Figure 7:**
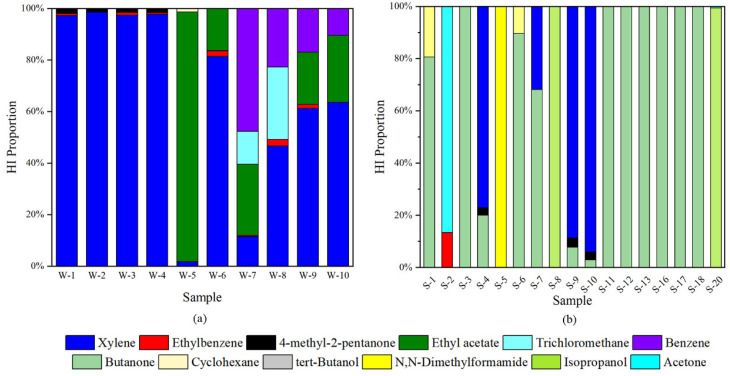
Proportions of the noncancer risks of volatile compounds emitted from white emulsion adhesives (**a**) and silicone adhesives (**b**).

Among the 21 compounds that we identified, 12 compounds were selected as the target VOCs for noncancer risk assessments, and 3 compounds were related to cancer risks. The toxicological parameters can be obtained from the Integrated Risk Information System and the Risk Assessment Information System of the USEPA database. The proportions of HI of each volatile compound in the white emulsion adhesive based on EC are shown in Figure 7a. As shown in Figure 7a, the average HI proportion of xylene was 65.73%, which made it a key volatile pollutant from the perspective of noncancer risk. In addition, ethyl acetate, benzene and trichloromethane played significant roles in the noncancer risks, with average HI proportions of 37.41%, 24.38% and 20.43%, respectively. The noncancer risks of white emulsion adhesives were mainly contributed by xylene, ethyl acetate, benzene and trichloromethane because their reference concentration (RfC) values were much lower than those of the other substances and because their chemical concentrations were also very high.

In terms of cancer risks, the proportions of R of each volatile compound in the white emulsion adhesive are shown in Figure S2. Only the R proportions of ethylbenzene, trichloromethane and benzene are determined in this study, which dominated the overall cancer risks in the gaseous emissions from the white emulsion adhesives. Specifically, ethylbenzene, trichloromethane and benzene contributed 65.85%, 77.22% and 46.15%, respectively. As a result, ethylbenzene, trichloromethane and benzene can be identified as the key toxic volatile pollutants due to their high R contributions. These results indicated that adhesives may have adverse health effects on construction workers and indoor residents. The improvement of adhesive formulas to reduce health risks deserves more attention.

For the silicone adhesive samples, the HI proportions of 8 compounds from 17 samples are illustrated in Figure 7b. The compounds related to the noncancer risks in silicone adhesive samples were different from those in white emulsion adhesives. Butanone oxime, butanone and ethanol were the three substances with the highest detection frequencies, but only butanone could be traced for the toxicity parameters. In addition to butanone, xylene (72.88%), isopropanol (99.69%) and *N*,*N*-dimethylformamide (100%) played important roles in the cumulative noncancer risks. For the cancer risks, only the R value of ethylbenzene in sample S-2 was available. Overall, the health risks of gaseous emissions from silicone adhesives were much lower than those of the white emulsion adhesives. 

From an integrated perspective of chemical emissions, odor and health risk contributions, xylene, ethylbenzene, ethyl acetate and benzene were identified as the key volatile pollutants emitted from the white emulsion adhesives, while butanone oxime, butanone and ethanol were the key volatile pollutants emitted from the silicone adhesives. The chemical compositions and emission factors of the VOCs emitted from adhesives have been well addressed in previous studies [34,39,40,41], while the current research emphasized that the key volatile pollutants causing odor nuisances and health risks may differ among different kinds of adhesives. This comprehensive methodology could be a promising approach to identify the key pollutants in complex gaseous emissions from adhesives and other building materials. Additionally, compared to the evaluation of indoor air mixtures, this method provided delicate knowledge of key volatile pollutants emitted from different emission sources, offering informative strategies to precisely reduce the related odor nuisances and health risks.

## 3. Materials and Methods

### 3.1. Sample Description

The adhesives used in the present study were provided by the China Building Materials Research Institute, China Building Material Test & Certification Group. According to the use of adhesives, we selected 2 types; namely, white emulsion adhesives and silicone adhesives, from 30 different brands and manufacturers. A total of 10 white emulsion adhesive samples and 20 silicone adhesive samples were selected.

### 3.2. Gas Preparation

The adhesive samples were prepared by painting a certain amount of adhesive on a 2×5 cm glass plate. According to the test requirements “Test Methods for the Release of Hazardous Substances from Indoor Materials and Articles Building Material Products” issued by China Building Material Test & Certification Group Corporation, the adhesive sample load rate was set at 0.4 m^2^/m^3^, the product usage was 100 g/m^3^ and the emitting time was 2 h. The adhesive sample was placed in a sealed gas emission chamber (total volume: 2L) immediately after weighing. There was a removable chassis with a sampling port on the bottom of the chamber, through which the adhesive sample was placed inside. During the tests, the chassis was screwed tightly onto the chamber and the sampling port was sealed to ensure gas tightness. After the adhesive sample was emitted for 2 h, the sampling port on the chassis was opened and the top cap of the chamber was pressed down at a constant speed by a motor, squeezing the gas sample in the chamber to flow out from the sampling port (Figure S1). A certain amount of the emitted gas sample was collected from the sampling port by a gastight syringe for chemical analysis. 

Moreover, two samples from white emulsion and silicone adhesives were selected to investigate the effect of pre-emission time on the results. Pre-emission was conducted before placing adhesive samples in the emission chamber to simulate different operation methods of adhesives during indoor decoration. Then, the samples were transferred to the emission chamber to prepare gas samples using the same method as that for the previous 30 adhesive samples.

### 3.3. Chemical Analysis

Gas chromatography-mass spectrometry (GC/MS, TRACE1300-ISQLT, Thermo Fisher Scientific, Waltham, MA, USA) was used to analyze the compositions and chemical concentrations of the volatile compounds in the gas sample. Chemical analysis was performed on a GC/MS system coupled with a DB-WAX-52CB (30 m × 0.25 mm × 0.25 um) capillary column. A 0.7 mL gas sample was injected into the GC/MS system using a gastight syringe. The temperature of the GC/MS inlet was set as 250 °C. The initial temperature of the oven was 50 °C, which was maintained for 3 min, then increased to 155 °C at a rate of 15 °C/min, and held for 10 min. He (99.999%) was used as the carrier gas at a flow rate of 1.2 mL/min. The compounds separated by GC were then analyzed by MS over the mass-to-charge ratio range of 40-350 amu. The temperatures of the ion source and transfer line were both set at 250 °C. The detected compounds were identified by comparing the corresponding mass spectra with the standard mass spectral library. Quantification of the detected compounds was conducted by the external standard method.

### 3.4. Odor Analysis

A dynamic dilution olfactometer (AC’SCENT International Olfactometer, St. Croix Sensory, Stillwater, MN, USA) was used to determine the odor thresholds of the detected volatile compounds. Odor threshold (*C_OT_*) values were needed to assess the odor contribution of the detected compounds. For the compounds whose *C_OT_* values were not available in the literature, we measured their *C_OT_* with a dynamic olfactometer (AC’SCENT, USA). The procedure of the olfactometric analysis followed the instructions of the Forced-Choice Ascending Concentration Series Method, which meets both ASTM E679-04 (ASTM-E679-04, 2011) and EN 13725:2003 (EN13725, 2003) standards [50].

The odor activity value (*OAV*) method was used to assess the odor contribution of the individual volatile compounds to the overall gaseous mixture. The chemical concentrations of volatile compounds were converted into *OAVs* by coupling with *C_OT_* according to Equation (1), and the sum of odor activity values (*SOAVs*) was calculated according to Equation (2). The *OAV* proportion (*P_i_*) was calculated by Equation (3) to assess the odor contribution of individual compounds to the overall gaseous mixture emitted from the adhesive samples.

(1) $$OAV=\frac{C}{C_{OT}}$$

(2) $$SOAV=\sum OAV_{i}$$

(3) $$P_{i}=\frac{OAV_{i}}{SOAV}$$

### 3.5. Data Analysis

PASW Statistics software (version 18.0) (IBM, Hong Kong, China) and Origin software (version 2018) (OriginLab, Northampton, MA, USA) were used for data processing.

## 4. Conclusions

This research characterized the volatile compounds emitted from 30 adhesive samples. A total of 21 volatile compounds were detected and quantified, of which xylene and butanone oxime were the most concentrated compounds in white emulsion adhesives and silicone adhesives, respectively. White emulsion adhesives and silicone adhesives could be divided into two main groups depending on their compositions and concentrations by cluster analysis. Moreover, based on the odor and health risk contributions, the key volatile pollutants of the two adhesives were identified. In addition to enriching the existing literature on volatile compound emissions from building materials, this research presented a methodology for determining the key volatile pollutants from the integrated perspective of chemical emissions, odor and health risk contributions. In view of the large dosage of adhesives in interior decoration, such a characterization could provide knowledge on the mitigation of the odor and health risks caused by indoor air pollutants to decoration workers and residents.

## Figures and Tables

**Table 1:** Summary of the concentrations of compounds during VOC emission of adhesive samples (mg/m^3^) and the corresponding odor threshold value (mg/m^3^).

Categories	Compounds	Concentrations (mg/m^3^)					Odor Threshold Value (mg/m^3^)
		Mean	Max	Med	Min	Fre (%)	
White emulsion adhesives	4-Methyl-2-pentanone	10.35	55.34	0.66	0.25	100	0.7
	Ethylbenzene	10.27	16.76	10.82	2.32	70	0.73
	Xylene	42.70	125.99	36.88	0.76	100	0.68
	Ethyl acetate	21.47	40.61	17.54	5.67	50	3.1
	Trichloromethane	22.23	26.38	22.23	18.08	20	19
	Benzene	29.69	66.78	18.34	15.29	40	8.6
	Butanone	2.85	3.30	2.85	2.41	20	1.3
	Methyl acetate	7.35	18.52	3.61	0.89	70	5.1
	Cyclohexane	9.01	35.47	0.22	0.12	30	8.5
	Dibutyl ether	3.18	5.76	3.26	0.50	30	0.11
	Tert-Butanol	4.62	4.62	4.62	4.62	10	14
Silicone adhesives	Butanone oxime	668.34	2221.74	484.32	22.30	100	0.42
	Butanone	33.17	108.81	10.04	4.13	65	1.3
	4-Methyl-2-pentanone	6.95	10.28	7.36	3.22	15	0.7
	Ethanol	231.02	1132.35	98.69	2.59	70	0.99
	Isopropanol	79.30	128.33	79.30	30.27	10	65
	Butanol	26.41	46.72	26.41	6.09	10	0.11
	Ethylbenzene	0.18	0.18	0.18	0.18	5	0.73
	Xylene	4.75	10.03	4.34	0.30	20	0.68
	Butyl acetate	7.14	7.14	7.14	7.14	5	0.077
	Ethyl acetoacetate	41.34	41.34	41.34	41.34	5	0.025
	Methyl acetoacetate	360.00	360.00	360.00	360.00	5	0.062
	*N*,*N*-Dimethylformamide	1.89	1.89	1.89	1.89	5	0.79
	Acetone	33.03	37.10	33.03	28.96	10	10
	Cyclohexane	8.17	15.72	8.17	0.62	10	0.609

(Med: median, Max: maximum, Min: minimum, Fre: detection frequency).

## Data Availability

Not applicable.

## Supplementary Materials

The following supporting information can be downloaded at online, Figure S1: Structure and components of the sealed gas emission chamber; Figure S2: Proportions of the cancer risks of volatile compounds emitted from white emulsion adhesives; Table S1: Odor activity values (*OAVs*) of volatile compounds emitted from white emulsion adhesive; Table S2: Odor activity values (*OAVs*) of volatile compounds emitted from silicone adhesive.

## Sample Availability

Samples of the compounds are available from the authors.
