# Supplementary material for: Characterizing Key Volatile Pollutants Emitted from Adhesives by Chemical Compositions, Odor Contributions and Health Risks
Source: Molecules. 2022 Feb 8;27(3):1125. doi: 10.3390/molecules27031125 (PMC8839774; doi:10.3390/molecules27031125)

Supplementary Information

Characterizing key volatile pollutants emitted from adhesives by chemical compositions, odor contributions and health risks

Zixuan Zhao[1,2], Yipu Pei[3], Peng Zhao[d], Chuandong Wu[1], Chen Qu[1], Weifang Li[2], Yanjun Zhao[1], Jiemin Liu[1,*]

1 *School of Chemistry and Biological Engineering, University of Science and Technology Beijing, Beijing, 100083, China*; zzxmht@163.com (Z.Z.); wuchuandong@ustb.edu.cn (C.W.); quchen5626@163.com (C.Q.); zhaoyj@ustb.edu.cn (Y.Z.); liujm@ustb.edu.cn (J.L.)

2 *State Environmental Protection Key Laboratory of Odor Pollution Control, Tianjin Academy of Eco-environmental Sciences, Tianjin, 300191, China*; zzxmht@163.com (Z.Z.); lwf1919@163.com (W.L.)

3 *China Building Material Test & Certification Group Co., Ltd, Beijing, 100024, China*; peiyipu@ctc.ac.cn (Y.P.)

4 *Beijing Municipal Institute of Labor Protection, Beijing 100054, China*; zhaopeng@bmilp.com (P.Z.)

* Correspondence: liujm@ustb.edu.cn (J.L.); Tel.: (+86)-10-8237-6678; fax: (+86)-10-6233-2281

.

Figure S1. Structure and components of the sealed gas emission chamber.

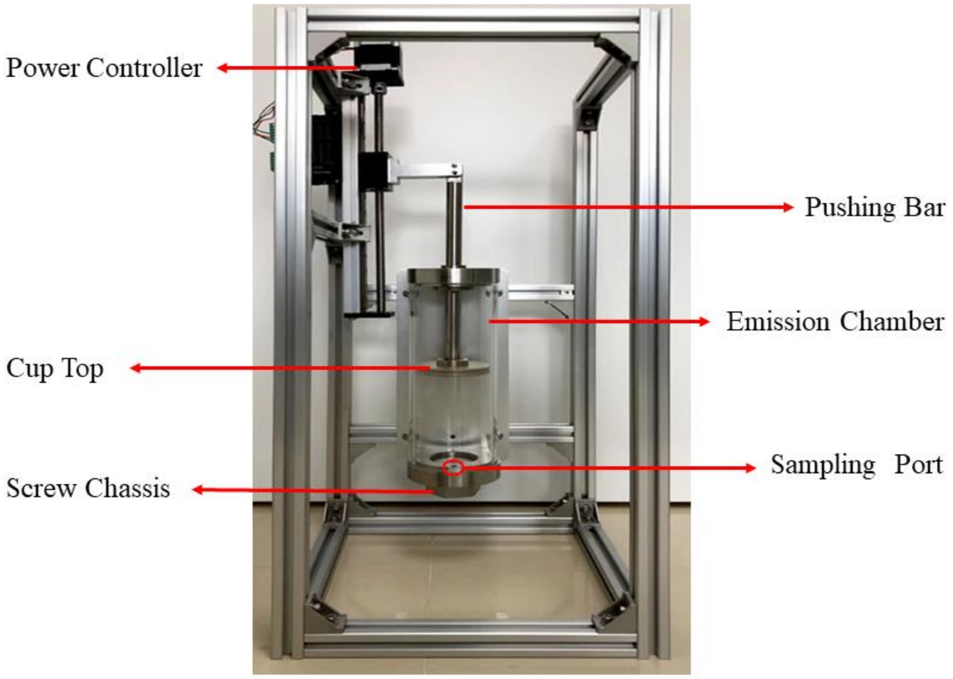

Table S1. Odor activity values (OAVs) of volatile compounds emitted from white emulsion adhesive.

	Xylene	Ethylbenzene	4-methyl-2-pentone	Ethyl acetate	Methyl acetate	Trichloromethane	Benzene	Butanone	Cyclohexane	tert-Butanol	Dibutyl ether
**S-1**	66.09	4.91	37.74								
**S-2**	10.94		4.47		2.03						
**S-3**	185.28	18.86	79.06							0.33	30.01
**S-4**	49.25	3.19	23.01		3.63						53.12
**S-5**	1.12		1.16	9.72					4.17		
**S-6**	59.22	14.82	0.43	1.83	0.68						
**S-7**	35.84	13.63	0.36	13.10	0.71	1.39	3.50				
**S-8**	45.05	22.96	0.55		2.70	0.95	0.52		0.029		
**S-9**	85.08	20.14	0.42	4.32	0.18		0.56	1.85	0.014		
**S-10**	90.09		0.75	5.66	0.178		0.35	2.54	0.024		4.64

Table S2. Odor activity values (OAVs) of volatile compounds emitted from silicone adhesive.

	Butanone oxime	Butanone	Ethanol	4-methyl-2-pentone	Ethylbenzene	Xylene	Butyl acetate	Ethyl acetoacetate	Methyl acetoacetate	N, N-dimethylformamide	Isopropanol	Butanol	Acetone	Cyclohexane
**W-1**	3206.54	54.60												1.85
**W-2**	53.08				0.25		92.66					424.72	0.34	
**W-3**	3934.30	83.70	175.38											
**W-4**	2806.39	52.26	294.51	4.59		4.02								
**W-5**	124.32		905.87							2.39				
**W-6**	1069.75	4.48	121.27											0.07
**W-7**	3154.77	47.09	66.32			0.44								
**W-8**	347.87							1653.41	5806.51		1.97			
**W-9**	5289.86	38.23		10.51		8.73								
**W-10**	3298.17	22.66	1143.79	14.68		14.75								
**W-11**	895.44	4.48	93.48											
**W-12**	1575.72	4.37	96.58											
**W-13**	719.55	4.39	2.62											
**W-14**	69.51											55.39		
**W-15**	113.19		102.80											
**W-16**	1121.43	4.53	25.58											
**W-17**	1219.32	3.18	22.38											
**W-18**	1543.74	7.72	73.65											
**W-19**	1184.85		142.79											

**W-20**	98.04	0.47	0.27

Figure S2. Proportions of the cancer risks of volatile compounds emitted from white emulsion adhesives.

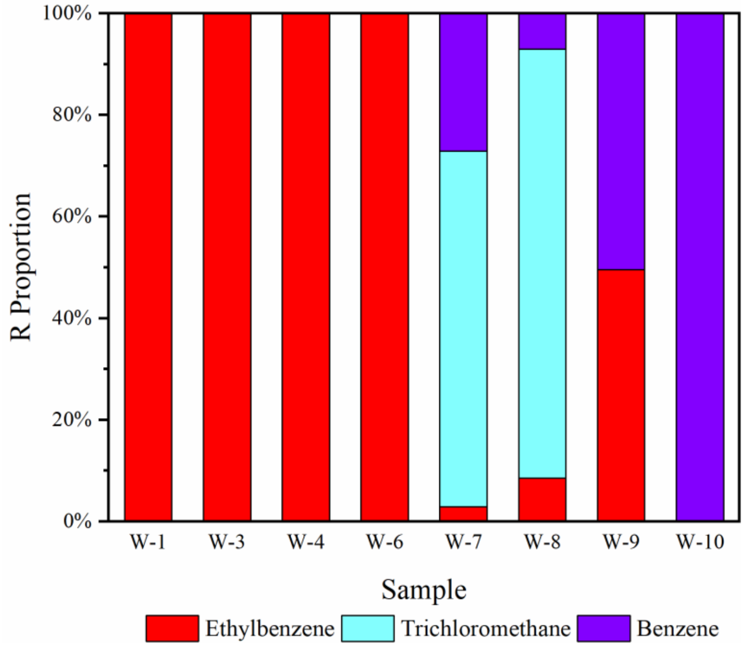

Supplement: Supplementary file 1 [file molecules-27-01125-s001.zip › molecules-1567807-supplementary.pdf]
